# Neural Representation of Scale Illusion: Magnetoencephalographic Study on the Auditory Illusion Induced by Distinctive Tone Sequences in the Two Ears

**DOI:** 10.1371/journal.pone.0075990

**Published:** 2013-09-23

**Authors:** Shinya Kuriki, Koichi Yokosawa, Makoto Takahashi

**Affiliations:** 1 Research Center for Science and Technology, Tokyo Denki University, Inzai, Japan; 2 Graduate School of Health Science, Hokkaido University, Sapporo, Japan; 3 Graduate School of Information Science and Technology, Sapporo, Japan; Radboud University Nijmegen, The Netherlands

## Abstract

The auditory illusory perception “scale illusion” occurs when a tone of ascending scale is presented in one ear, a tone of descending scale is presented simultaneously in the other ear, and vice versa. Most listeners hear illusory percepts of smooth pitch contours of the higher half of the scale in the right ear and the lower half in the left ear. Little is known about neural processes underlying the scale illusion. In this magnetoencephalographic study, we recorded steady-state responses to amplitude-modulated short tones having illusion-inducing pitch sequences, where the sound level of the modulated tones was manipulated to decrease monotonically with increase in pitch. The steady-state responses were decomposed into right- and left-sound components by means of separate modulation frequencies. It was found that the time course of the magnitude of response components of illusion-perceiving listeners was significantly correlated with smooth pitch contour of illusory percepts and that the time course of response components of stimulus-perceiving listeners was significantly correlated with discontinuous pitch contour of stimulus percepts in addition to the contour of illusory percepts. The results suggest that the percept of illusory pitch sequence was represented in the neural activity in or near the primary auditory cortex, i.e., the site of generation of auditory steady-state response, and that perception of scale illusion is maintained by automatic low-level processing.

## Introduction

**Figure 1:**
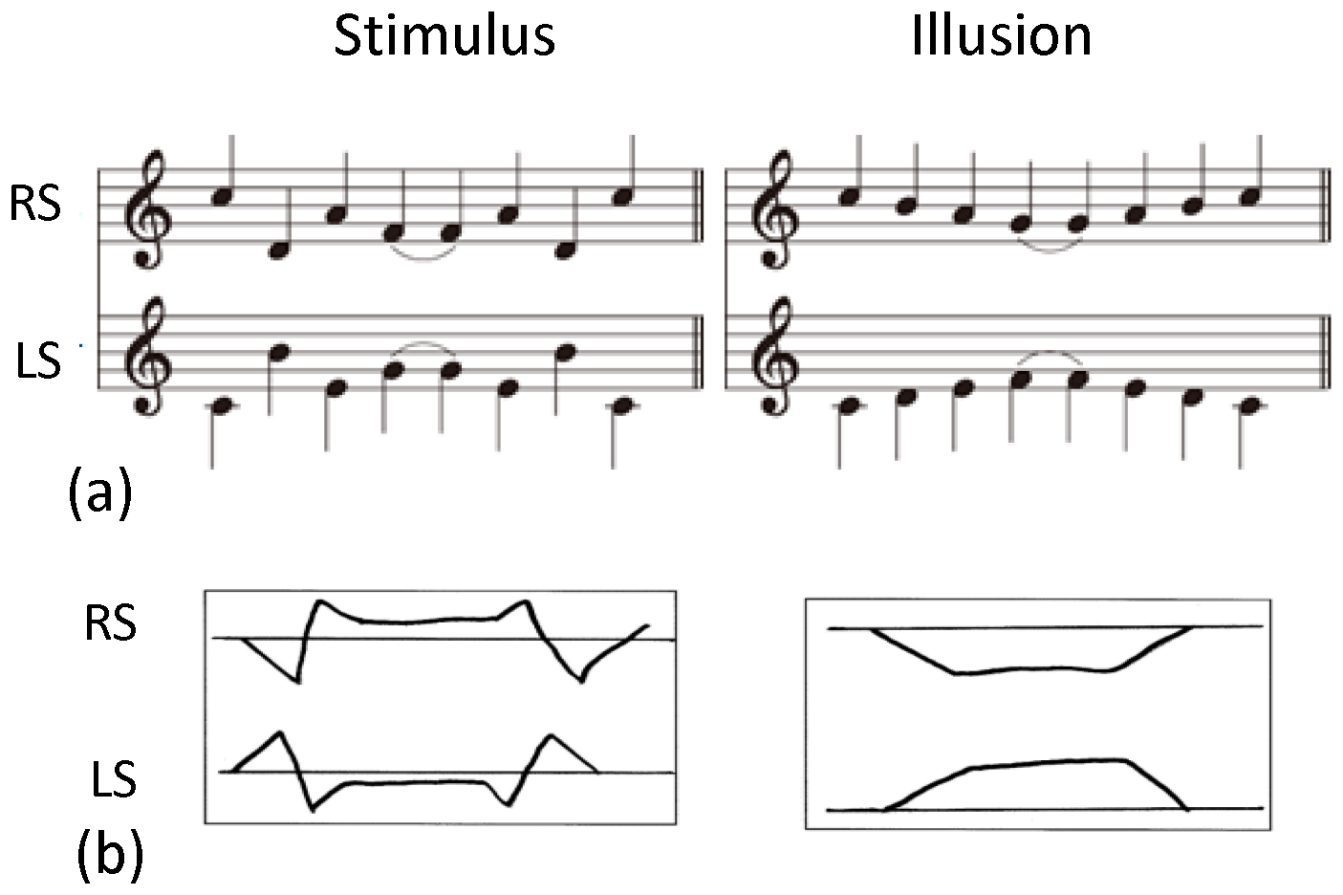
Musical notes and subjects' reports for stimulus and illusion tone sequences. (a) Stimulus tones consisted of distinct pitch sequences of right sound (RS) presented to the right ear and left sound (LS) presented to the left ear. Most of reported percepts of illusion were high pitch tones of the right-ear sound and low pitch tones of the left-ear sound. (b) Example of subjects' drawings of pitch contour they heard as right-ear and left-ear sounds, representing stimulus type and illusion type percepts.

The “scale illusion” is an illusory perception of auditory sounds that are delivered to listeners' right and left ears in distinctive pitch sequences but heard as altered-pitch streams in the two ears. In its stimulation paradigm [1], sequences of ascending and descending tones within a C-major scale are presented simultaneously, such that when a tone of the ascending scale is in one ear, a tone of the descending scale is in the other, and vice versa. The resulting tone sequences are discontinuous in pitch (Fig. 1a, left), but listeners tend to perceive illusory smooth contours distinctively in the right and left ears (Fig. 1a, right), which consist of the higher and lower halves of the scale. The illusory percepts are thought to be the result of grouping of scale tones, concurrently input from both ears, into high and low pitch streams localized to the right and left ears [1]. The neural mechanism responsible for the creation of illusory percepts should reside in the central nervous system because the two ears' tones are separated in the periphery. However, little is known about where the mechanism exists in the auditory system and what are the neural processes underlying the scale illusion. The scale illusion is an unsolved issue of binaural perception in the auditory system in which temporal information processing is crucial.

There have been few reports of neurophysiological studies on the scale illusion in humans, but some are reported on the octave illusion in which a pair of octave-spaced tones is presented to both ears repeatedly in alternation [2]. The most common illusory percept of the octave illusion is a single high tone in one ear, alternating with a single low tone in the other ear. The octave illusion has been studied with mismatch negativity of ERP (event-related potential) using infrequent, i.e., deviant, illusion-mimicking tones inserted in frequent illusion-evoking tones [3] and with transient MEG (magnetoencephalography) response of N1m evoked by binaural octave-spaced tones [4]. However, the results of those studies suggested controversial sites of the generator of illusory percept within and beyond the auditory cortex. The authors did not differentiate the responses to the right and left ears and thus could not take into account the interaction of the tones from the two ears. In a recent study by Lamminmäki et al. [5], auditory steady-state response (ASSR) was measured for continuous amplitude-modulated (AM) tones that were octave-spaced and delivered separately to the two ears, where the modulation frequency differed between the two ears. The ASSR was decomposed into the left- and right-ear responses from the tagged, i.e., differentiated, modulation frequencies of the AM tones. They observed suppression of ipsilateral response and enhancement of contralateral response to the dichotic tones of octave-spaced pitches and discussed the contribution of these binaural interactions to the illusory perception. However, they did not study the change in the suppression/enhancement of the response as the octave-spaced tones alternated between the two ears, which is important in the dynamics of octave illusion.

The frequency tagging had been attempted in visual [6] and auditory [7] responses of MEG and proved to be an effective method to discriminate two-channel sensory activities. In this study, we employed this method to examine MEG responses in the right and left hemispheres separately during the perception of scale illusion. We replaced each of the stimulus tones that comprised Deutsch's musical notes with AM tones of the same pitch, where modulation frequencies of the right and left AM tones were tagged with slightly different frequencies. Since this method relies on ASSR, instead of transient evoked responses to the stimulus tones, characteristics of ASSR should be taken into account in the approach to the neural representation of scale illusion.

Generation of ASSRs in the auditory system is related to the temporal processing of stimulus sound, being time-locked to the specific phase of the waveform of the sound. Animal studies have shown that phase locking to AM sound occurs along the auditory pathway from the peripheral auditory nerve to the medial geniculate body (MGB) of the thalamus, [8], [9], [10], [11], [12], decreasing the maximum responding frequency from the peripheral to central structures. In the primary auditory cortex (A1), phase-locked responses are observed at low AM frequencies below about 20 Hz [13], [14], [15], but stimulation by complex sounds evokes responses at higher frequencies up to 80 Hz [16]. Direct recordings from the MGB and auditory cortex have shown distinct ASSRs in a frequency range of 20–100 Hz of click stimulation [17]. In human ASSR of potential, cortical and subcortical origins at the thalamus and brainstem have been suggested from cross-modal stimulation [18], intracranial recording [19], patient study [20] and scalp topography [21]. Effects of noise on the scalp ASSR have suggested that high-frequency (80 Hz) ASSR contains component from the brainstem and 40-Hz ASSR contains component from the upper auditory system [22]. The inclusion of subcortical signals in the scalp ASSR is due to volume conduction of the potential through electrically conducting medium. In contrast, neural sources of the ASSR of MEG, which directly detects the magnetic field produced by intracellular currents, have been shown to be located in or near the A1 in the superior temporal plane [23], [24], [25], [26]. Low-frequency ASSR (10 Hz) of MEG has no inclusion of a brainstem component [27].

Given that frequency-tagged ASSRs could discriminate the auditory cortical activities related to the right- and left-ear inputs, characteristics of ASSR for briefly presented tones of less than 1 s in length should be established. This temporal resolution is deduced from a report that 500-ms period is most favorable for inducing illusory perception [28]. We have shown in a separate study [29] that the amplitude of ASSR elicited by short-duration (0.78 s) AM tones does not depend on tone frequencies between 440 and 990 Hz in about one octave range but is determined in a proportional manner by the sound pressure level of the tone. These characteristics served as the basis in the processing of recorded MEG signals in the present study. The aim of this study was to clarify the cortical neural representation of scale illusion to reveal whether the pitch sequence of stimulus tones that induce illusory percepts is encoded as it is presented or as it is perceived. We found that the time course of the amplitude of ASSR was significantly correlated with the pitch contour of the illusory percept in subjects who reported scale illusion, suggesting that the percept of illusory pitch sequence is represented in the auditory cortical activity.

## Materials and Methods

### Subjects and stimuli

Fourteen subjects participated in the experiment. Among them, the data of two subjects (S1, S3) were discarded because of the existence of noise-contaminated channels in the temporal area in MEG recordings. The rest of 12 subjects had a mean age (SD) of 21.9 (1.3). They had experience in practicing/playing musical instruments for a mean period of 10.6 (5.2) years. This is based on a previous report that musical experience facilitated the perception of scale illusion in listeners [30]. All but one of the subjects were right-handed. All the subjects gave written informed consent to participate in the study after receiving an explanation of MEG recording. The study was approved by the Ethics Committee of School of Medicine, Hokkaido University, in which experiments were carried out.

The stimulus sound consisted of a series of eight amplitude-modulated (AM) sinusoidal tones, each with a length of 745 ms, connected without intermission. The AM tones in the series had various carrier frequencies to follow the pitch of the notes given by Deutsch [1]. We used an equally-tempered C major scale with a fundamental pitch (A4) of 435 Hz as in [1]. The carrier frequency of the tones ranged in one octave from 517.3 Hz (C5) to 1034.6 Hz (C6). Stimulus sounds of two different sequences of carrier frequency, as the notes in Fig. 1a left, were prepared for the right and left ear stimulation with separate modulation frequencies of 43.75 and 35.80 Hz (abbreviated as 44 and 36 Hz), respectively. The modulation depth was 100%. Such AM sounds were expected to evoke steady-state responses, which would be differentiated by their component frequencies into right-sound and left-sound responses. Each of the 745-ms tones in the sequence consisted of 27 AM waves in the right and 33 AM waves in the left sounds, where the transition of carrier frequency at the boundary to the adjacent tone occurred at the time of zero amplitude in the AM waves.

**Figure 2:**
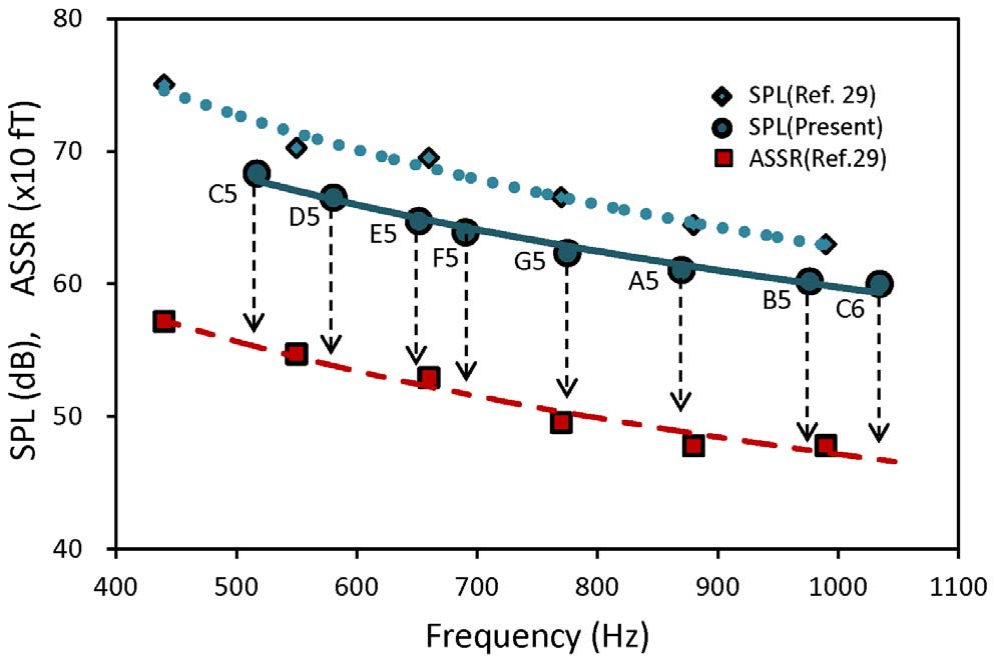
Sound pressure levels (SPL) in dB of stimulus AM tones used in a separate [29] and present studies. Dotted and solid lines represent approximation curves of logarithmic dependence on the carrier frequency. Also shown are magnitudes of ASSR elicited by the AM tones in [29], from which the variation of ASSR magnitude with the pitch of C-major key was estimated (vertical broken lines).

Stimulus-sound signals were composed in a PC at a sampling rate of 44.1 kHz. Amplitudes of AM tones were arranged to be monotonically higher for lower carrier frequency. The sound delivery system consisted of two-channel amplifier and equalizer connected to separate transducers, plastic tubes and ear pieces. The frequency characteristic of the sound delivery system was within 2 dB from 20 Hz to 4 kHz. The sound pressure level (SPL) of the stimulus sound was adjusted using a sound meter at the earpiece to be 60 dB at 1 kHz, being equal for the right and left channels. Fig. 2 shows the SPL values of the AM tones for different carrier frequencies, which correspond to the notes of C-major scale, where the SPLs of the short AM tones used in the separate study [29] are also shown. Assuming a logarithmic variation (dotted line in Fig. 2) of the SPL in dB with carrier frequency of the AM tones used in [29], calculated SPLs for the C-major pitches in this study correlated well with a coefficient of 0.989 (p<0.001, t-test; t(4) = 13.5). Thus, the frequency dependence of the tone intensities in the two studies agreed well. The magnitudes of ASSRs that would be obtained for the stimulus tones in this study were estimated at different pitches of C-major key, as indicated by vertical broken lines in Fig. 2. Fifty sets of the sequence of eight AM tones were connected without intermission, lasting for 5 min, and presented to the subject in a single session of MEG recording. A rest period for a few minutes was inserted after the MEG recording. Each subject underwent four sessions of the recording.

### Behavioral measurements and MEG recordings

Behavioral measurements were carried out to inspect the subjects' percept of dichotic stimulus sounds. The subjects were instructed to write down pitch contours of the sound they heard in the right and left ears, while listening to the sequence of eight stimulus tones repeatedly. The measurements were performed for the subjects seated and covered with the whole-head MEG sensors immediately before each of the four recording sessions, using the same tones with stimulus sounds. The subjects' percept of either illusory or stimulus sequence was judged from the outlines of the pitch they reported (e.g., Fig. 1b). For the combination of tone sequences and ears used in this study, most of the reported illusory percepts were high-pitch tones occurring in the right ear and low-pitch tones in the left ear (Fig. 1a).

MEG recordings were performed using a helmet-shaped SQUID system (Elekta- Neuromag, Helsinki), which had 76- and 19-channel sensors (magnetometers) arranged on a lower layer and upper layer, respectively. The two sensor layers formed concentric spherical surfaces separated by 4 cm. The lower layer of 76 channels, with an inter-channel spacing of about 2.5 cm, was used in this study. Subjects were instructed to keep attention equally to the sounds in the two ears, i.e., not to attend selectively to the sound in a one ear. The recorded signals were bandpass-filtered to 0.1–100 Hz and sampled at 600 Hz. Averaging of the sampled signals was carried out across 50 epochs, where a single epoch corresponded to the sequence of eight tones, in each of the four recording sessions using a trigger time-locked to the beginning of the tone sequence. Signals exceeding 3 pT in amplitude were discarded during the averaging. Single epochs of the averaged signals included 6-s (0.745×8) time period.

### Data analysis

Before analyzing MEG data, the four recording sessions for each subject were classified into illusion and stimulus sessions from the results of the behavioral examination. Subjects also reported other schemes of percept such as a mixture of illusion- and stimulus-tone sequences split in the two ears. We selected the sessions in which unambiguous percepts of illusion and stimulus were confirmed. Two to four sessions of either percept were determined in individual subjects. The epoch signals were averaged across the selected sessions and decomposed into the ASSR components of two modulation frequencies of 36 and 44 Hz by wavelet analysis. We used Morlet mother-wavelet function with an m-factor (width) of 14, which had been determined in a simulation study using different m-values to discriminate sinusoidal waves of the two modulation frequencies. The wavelet analysis yielded the amplitude and phase of the ASSR as functions of time, from which the amplitude was used in subsequent processing. Ten MEG channels that exhibited larger ASSR amplitudes were selected in each of the right and left posterior-temporal areas to represent the responses in the right and left hemispheres, and the amplitudes of those channels were converged by averaging. Finally, the mean amplitude of ASSR during the tone length of 754 ms was calculated; this mean amplitude is referred to as the magnitude of the ASSR, hereafter. We thus obtained four components of ASSR that included the 44 Hz response, i.e., the response to the left sound (LS), and the 36 Hz response to the right sound (RS) in the right hemisphere (RH) and in the left hemisphere (LH). Each component consisted of a series of magnitudes of ASSR responding to eight stimulus tones differing in carrier frequency. It can be regarded as a wave of ASSR by connecting the magnitudes with a line.

**Figure 3:**
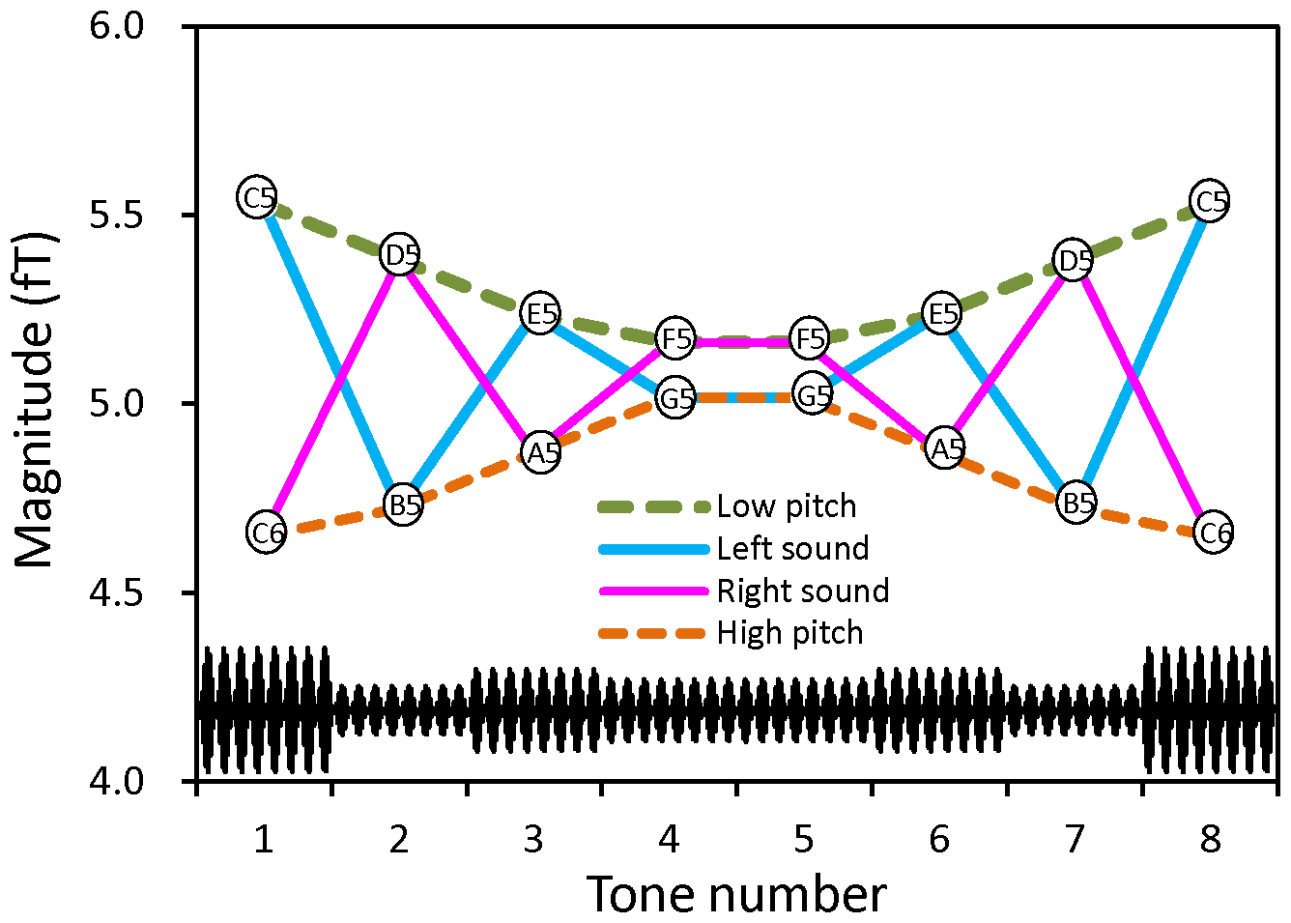
Estimated magnitude of ASSR for sequential AM tones that follow the scale notes in Fig. 1, i.e., right and left stimulus sounds and low-pitch and high-pitch illusory sounds. These stimulus type and illusion type variations of magnitude served as regressors of multiple regression analysis. A waveform of schematic stimulus tones is also shown for the “left sound”, where the amplitude of various-pitch tones determined the magnitude of ASSR.

Using the dependence of the magnitude of ASSR on the pitch of C-major key, as estimated in the process shown in Fig. 2, we calculated how the ASSR magnitude would vary as a function of the tone number for the pitch sequences of stimulus and illusion percepts. The results yielded stimulus (STM) type variations of the pitch sequences of LS and RS and illusion (ILL) type variations of the high-pitch and low-pitch sequences (Fig. 3). These STM type and ILL type variations were used as regressors of multiple regression analysis of ASSR. We carried out multiple regression analysis to dissolve the ASSR into the constituents of two types of variation, assuming the coexistence of responses to illusory and stimulus pitch sequences. Here, the ILL type and STM type regressors had a correlation coefficient of −0.354 between them, which was not significant (p>0.2, t-test). Therefore, the dissolved partial coefficients of these regressors are thought to be unequivocal. In the process of analysis, we normalized each component of ASSR in individual subjects by its grand mean magnitude across all subjects to compensate intersubject variability of the magnitude of ASSR. The regressors were also standardized to have a dispersion of 1 with zero mean. As the ILL type regressor, high-pitch and low-pitch sequences were used as default for the RS and LS components of ASSR, respectively. Consequently, we obtained a set of partial regression coefficients of the STM and ILL type variations for each component. When the obtained regression coefficients that had significantly nonzero values (p<0.05, t-test) were negative, we altered the ILL type regressors to those of low-pitch and high-pitch sequences for the RS and LS components, respectively, and recalculated the regression coefficients. As for the regression coefficients of STM type variations, significantly negative values were not observed.

## Results

### Behavior

Table 1 summarizes the results of subjects' percepts in the four sessions of behavioral measurement. Here, “ILL” means the normal illusion in which high-pitch tones were perceived in the right ear and low-pitch tones were perceived in the left ear. “R-ILL” is the percept of reversed illusion in which high- and low-pitch tones were perceived in the left and right ears, respectively. In “STM”, the perceived tones in the two ears agreed with the RS and LS of stimulus, while the RS and LS were perceived in the ears of the reversed side in “R-STM”. Subjects also reported “MIX”, i.e., a mixture of ILL and STM in the two ears, and “P-ILL” in which percepts of illusion were ambiguous in one ear. In one subject (S02), measurements in two sessions were missing for a technical reason. We selected the sessions of unambiguous percept of ILL or STM in individual subjects, and ASSR signals were averaged across the selected sessions, as described before. Data in the other sessions indicated by italics in Table 1 were discarded. We then classified the subjects into LL group having selected ILL sessions (upper half of Table 1) and STM group having selected STM sessions (lower half) with six subjects in each group. The ASSR signals of these groups were analyzed separately. The mean numbers of the selected sessions were 3.17 and 2.80 in the ILL and STM groups, respectively, with no significant difference in a t-test.

**Table 1:** Summarized results of subjects' percepts in the four sessions of behavioral measurements.

Subject	Session				Selected
	1st	2nd	3rd	4th	Sessions
S10	ILL	ILL	ILL	ILL	4
S13	ILL	ILL	ILL	ILL	4
S07	ILL	ILL	*R-ILL*	ILL	3
S12	ILL	ILL	*R-ILL*	*R-ILL*	2
S09	ILL	*MIX*	ILL	ILL	3
S06	ILL	ILL	*STM*	ILL	3
S08	*ILL*	*P-ILL*	STM	STM	2
S05	*P-ILL*	*MIX*	STM	STM	2
S04	*MIX*	STM	*R-STM*	STM	2
S14	STM	STM	STM	STM	4
S11	STM	STM	STM	STM	4
S02	STM	*not measured*		*STM*	2

MEG data shown by italic letters were not used in subsequent analysis. ILL =  normal illusion, R-ILL =  reversed illusion, P-ILL =  partial illusion, MIX =  mixture of illusion and stimulus, STM =  stimulus.

### Grand mean ASSR

**Figure 4:**
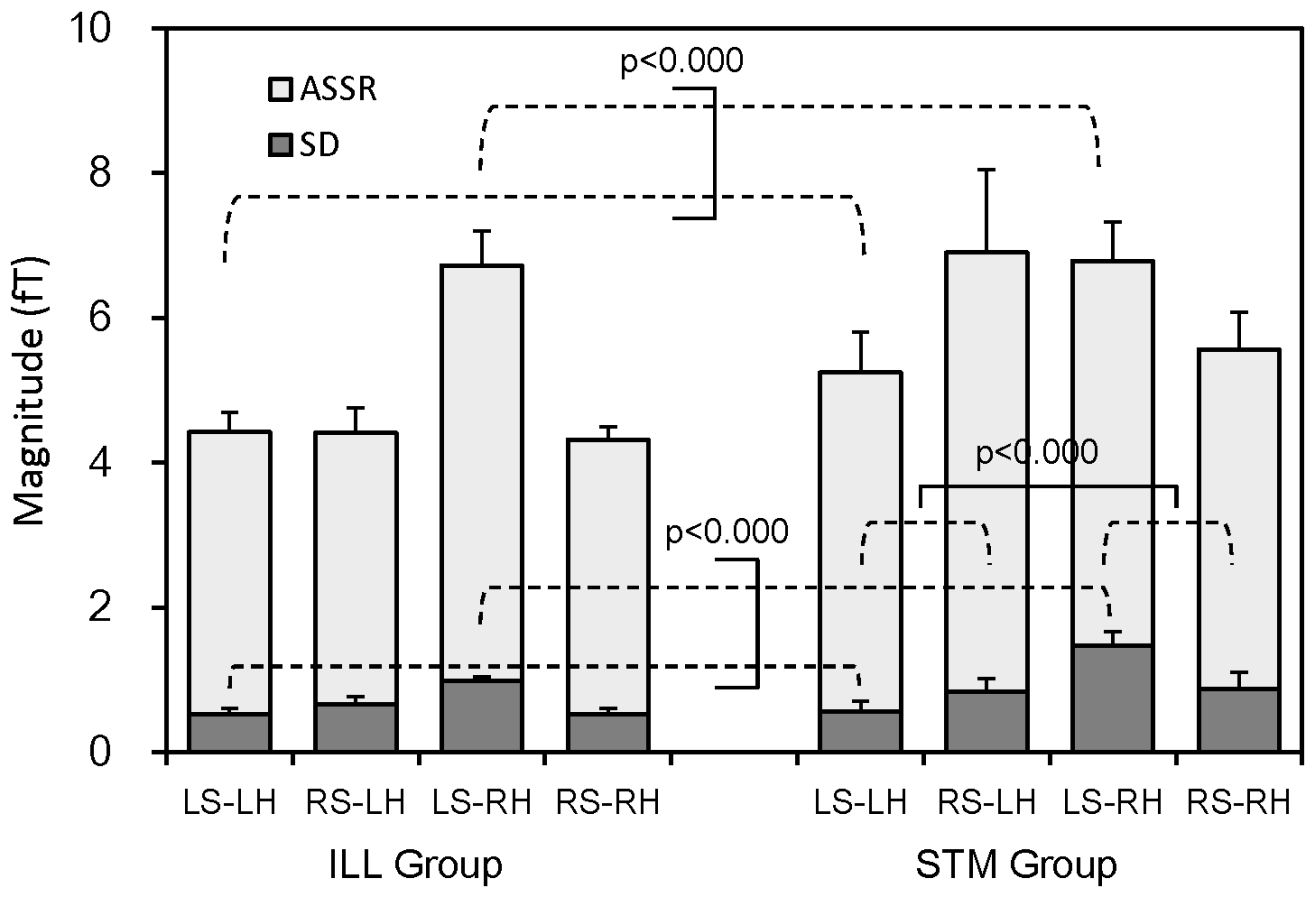
Grand-mean magnitude across stimulus tones and subjects of the ASSRs elicited by left and right sounds (LS and RS) in the left and right hemispheres (LH and RH). Results obtained for illusion (ILL) and stimulus (STM) groups are separately shown. Subject-mean of the standard deviation (SD) across stimulus tones of the ASSR magnitude is also shown. Note that the magnitude of ASSR is represented by the height of columns from zero line. Vertical bars indicate standard errors (SE) across subjects in each group (n = 6).

To examine overall features of ASSR, the grand mean magnitude of ASSR across eight stimulus tones and subjects was calculated. The results are shown in Fig. 4 for the four response components, i.e., LS and RS responses in the LH and RH, of ILL and STM groups. Also shown is the subject-mean of standard deviation of the ASSR magnitude across stimulus tones, which may correspond to the mean deflection of ASSR magnitude as the pitch changed from the first to eighth tones. Three-way ANOVA with factors of sound (LS/RS), hemisphere (LH/RH) and group (ILL/STM) revealed interactions of the sound and hemisphere for both the ASSR (p<0.0003, F(1,10) = 29.4) and standard deviation (p<0.002, F(1,10) = 19.1). Simple main effects showed that the ASSR and standard deviation elicited by the LS were greater in the RH than in the LH (p<0.0001, F(1, 20) = 22.7 for the ASSR and p<0.0000, F(1,20) = 45.0 for the standard deviation). This difference corresponds to the widely accepted contralateral dominance of auditory evoked responses, which was observed only for the stimulus sound to the left ear. There was an interaction for the standard deviation between the group and hemisphere. A simple main effect showed that the standard deviation in the STM group was greater in the RH than in the LH (p<0.0002, F(1,10) = 33.2).

**Figure 5:**
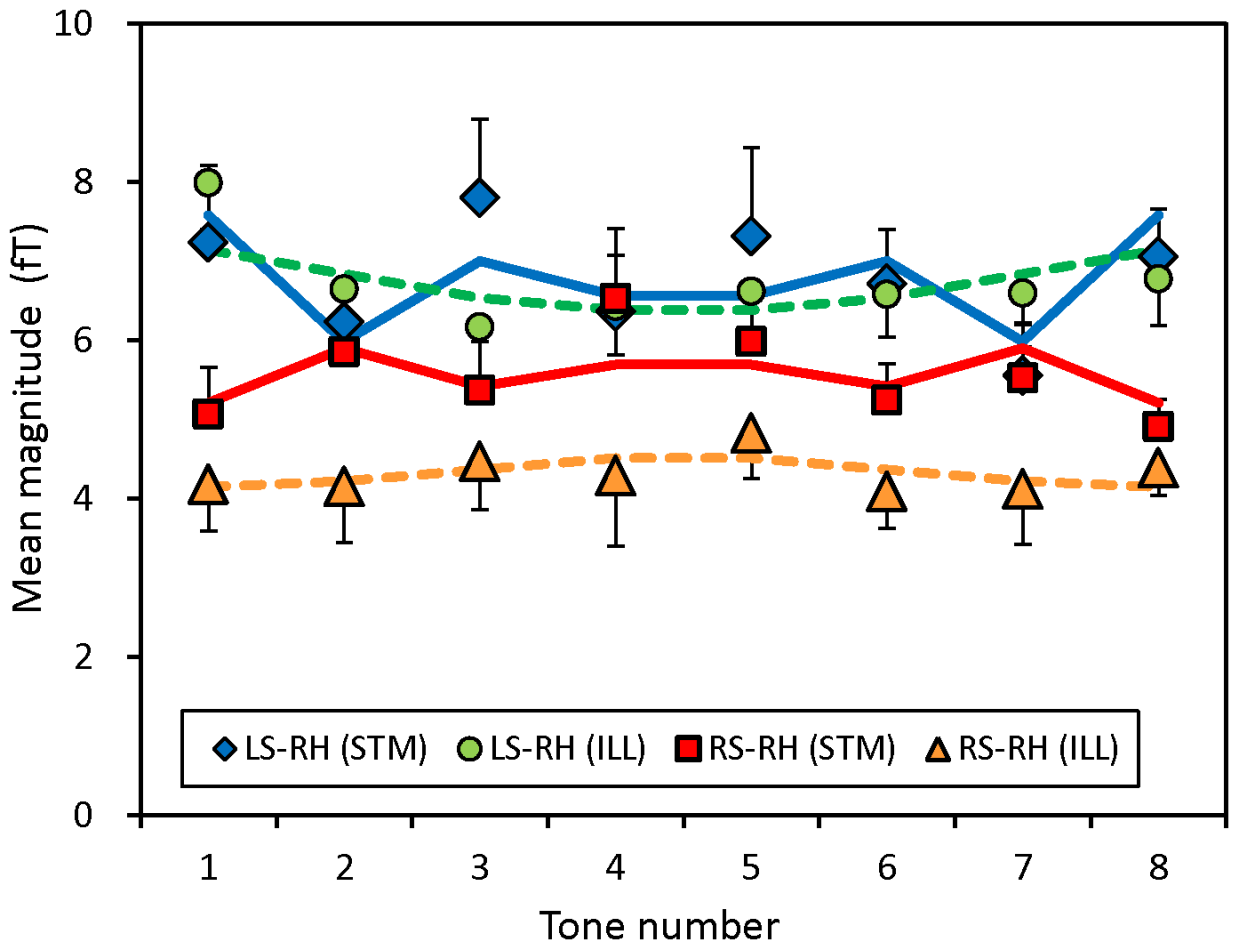
Mean magnitude across subjects of the ASSR of LS (contralateral) and RS (ipsilateral) responses in the RH as functions of stimulus-tone number. Results are shown by symbols for ILL and STM groups (n = 6 in each group), where SE bars are indicated either in the positive or negative direction to avoid overlapping. Calculated dependence is also shown as connected lines that were obtained using single ILL type (broken line) or STM type (solid line) variation of the ASSR (Fig. 3) and multiplying by an appropriate factor to fit the measured values. The calculated values were omitted for clarity.

Mean magnitudes of ASSR across subjects, as functions of tone number, are shown in Fig. 5 for the contralateral (LS) and ipsilateral (RS) responses in the RH. Here, observed values are indicated by different symbols. Calculated dependence is also shown as the waveform that was obtained using single ILL type (broken line) or STM type (solid line) variation of the ASSR (Fig. 3) and multiplying by an appropriate factor to fit the measured values. Crude matching of the calculated dependence indicates that the ILL type variation existed in the ILL group and the STM type variation existed in the STM group, but it also suggests that the mean ASSR consisted of a mixture of the two types of variation. However, multiple regression analysis using the subject-mean ASSR magnitudes did not give variability among subjects and thus did not allow for statistical evaluation for the calculated regression coefficients based on across-subjects variations.

### Regression coefficients

**Figure 6:**
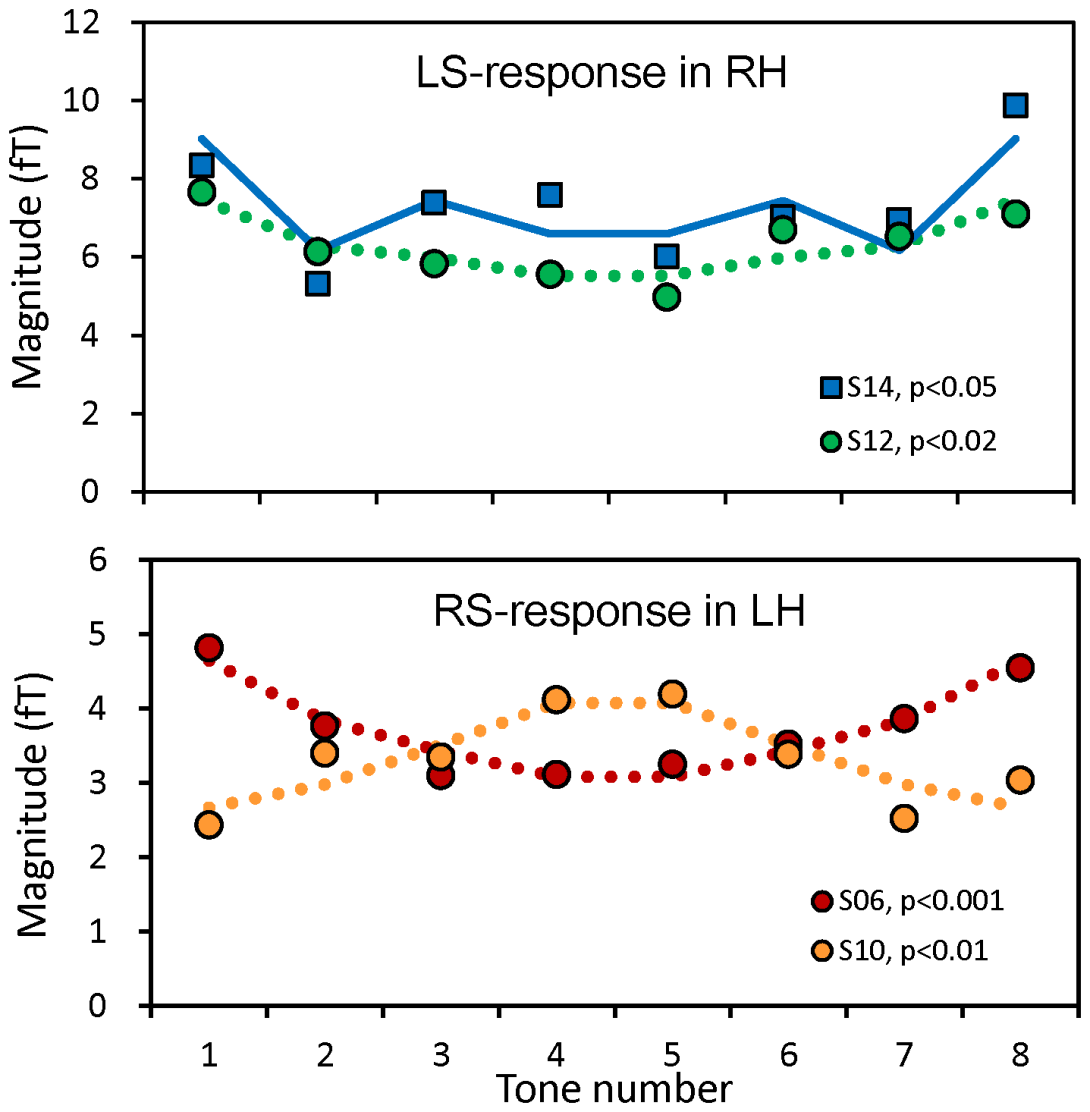
Magnitudes of ASSR as functions of tone number observed in (a) LS response in the RH and (b) RS response in the LH. Subjects belonged to the ILL (S12) and STM (S14) groups in (a) and to the ILL group (S06, S10) in (b). P-values are the significance levels of STM-type (square symbols) and ILL-type (circle symbols) regression coefficients obtained from multiple regression analysis. Synthesized variations using the regression coefficients are shown by the lines connecting calculated values.

We performed multiple regression analysis for the magnitudes of ASSR in individual subjects to decompose the ASSR into constituents of ILL type and STM type variations. Table 2 summarizes the obtained results of partial regression coefficients (*B*_i_) of ILL and STM type regressors (simplified as ILL type and STM type coefficients) for the response components of LS/RS-LH/RH. The value of *B*_i_ was small or even negative for the responses having erratic variation due to superimposed noises. The *B*_i_ values that were found to be significantly nonzero at a level of p<0.05 or better in the t-test are shown in bold letters. Examples of the variation of ASSR magnitude, for which significant coefficients were obtained, are shown in Fig. 6a for the LS response in the RH. In this response component, a subject (S12) in the ILL group and other subject (S14) in the STM group (S14) exhibited significant ILL type and STM type coefficients, respectively. Significant coefficients of reversed ILL type variations were also observed in LS and RS responses of one subject (S06) in the ILL group and two subjects (S04, S08) in the STM group (underlined in Table 2). In those subjects, the values of the ILL type coefficient were determined using high-pitch variation for the LS response and low-pitch variation for the RS response. Fig. 6b shows examples of the variation of ASSR magnitude having significant coefficients of normal- (S10) and reversed-illusion type (S06) variations. Clearly different (reversed) variations were observed for the same response component of the RS response in the LH. Synthesized dependence using those coefficients, shown by dotted lines, fitted well to the observed magnitudes.

**Table 2:** Illusion type and stimulus type partial regression coefficients decomposed by multiple regression analysis for the ASSRs consisting of left- and right-sound responses in the left and right hemispheres (LS/RS - LH/RH).

Illusion Group										
	Illusion type coefficient					Stimulus type coefficient				
Subject	LS-LH	RS-LH	LS-RH	RS-RH	C-Index	LS-LH	RS-LH	LS-RH	RS-RH	C-Index
S13	0.04	0.00	**0.56**	0.01	0.99	−0.09	0.00	0.37	−0.18	0.16
S10	0.15	**0.57**	0.23	0.17	1.00	0.16	0.01	−0.53	−0.09	−0.57
S07	**0.43**	−0.12	0.32	−0.15	0.48	−0.19	−0.02	0.50	0.09	0.47
S12	0.00	−0.10	**0.66**	0.18	0.78	0.40	0.52	0.21	−0.15	0.77
S09	0.32	−0.08	0.34	0.25	0.84	−0.15	0.43	−0.47	−0.03	−0.21
S06	0.04	**0.58**	0.40	−0.13	0.78	0.13	−0.09	0.55	0.06	0.78
Mean	0.16*	0.14	0.42***	0.05	0.81***	0.04	0.14	0.10	−0.05	0.24
Abs. Mean	0.16	0.24	0.42	0.15	0.81	0.19	0.18	0.44	0.10	0.49

Stimulus Group										
S08	0.24	−0.49	**0.69**	−0.01	0.30	0.17	−0.10	0.12	−0.07	0.26
S05	0.01	0.55	−1.02	0.45	−0.01	−0.20	−0.17	0.46	0.49	0.43
S04	−0.23	−0.09	**1.14**	**0.74**	0.71	0.22	0.21	0.43	−0.25	0.55
S14	0.08	−0.08	0.34	0.15	0.76	0.31	**0.69**	**0.98**	**0.73**	1.00
S11	0.03	**0.67**	**0.96**	**0.64**	1.00	0.29	0.12	**0.65**	0.11	1.00
S02	0.13	0.27	0.38	0.33	1.00	−0.16	−0.34	0.62	0.29	0.30
Mean	0.04	0.14	0.41	0.38**	0.63**	0.10	0.07	0.54***	0.22	0.59***
Abs. Mean	0.12	0.36	0.76	0.39	0.63	0.22	0.27	0.54	0.32	0.59

“Abs. Mean” is the mean of absolute values. “C-Index” is given by 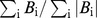, where *B*_i_ is the coefficient and i = 1 to 4. Values in bold letters indicate significantly positive (p<0.05 or better) coefficients. Underlined subjects exhibited coefficients of reversed-illusion type variation. Significance level of “Mean”:

*p<0.05,

**p<0.02,

***p<0.005.

The mean coefficients across subjects in Table 2 were significantly positive in some response components, in which p-values of the t-test are indicated by asterisks. The results of these significant mean coefficients indicated that ILL type variation existed in the responses of the ILL group and that both ILL type and STM type variations existed in the responses of the STM group. The “C (consistency)-index” is the sum of ILL type or STM type coefficients of four response components normalized by the sum of absolute coefficients, i.e., 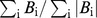, i = 1 to 4. This index indicates the extent to which the ILL type or STS type variation of the ASSR was established among the response components varying in sounds (LS/RS) and hemispheres (LH/RH). It would be 1 when ILL type coefficients consisted of low-pitch variation of the LS response and high-pitch variation of the RS response, vice versa for reversed illusion, and when STS type coefficients consisted of LS and RS variations of the LS and RS responses, respectively. It becomes small or even negative when some of the coefficients have negative values. It was found that the mean C-index across subjects was significantly positive for the ILL type coefficient in the ILL group and for ILL and STM type coefficients in the STM group. The subjects (S10, S13) that reported unambiguous illusion percepts in all of the four sessions (Table 1) had highest C-index values of 1 for the ILL type coefficient, together with significantly nonzero ILL type coefficients (p<0.05 or better) in the contralateral responses of (LS-RH)/(RS-LH) components. The mean multiple correlation coefficient, which indicates the matching of the combination of ILL type and STM type regressors to the observed ASSR variation, was 0.89. Likewise, the two subjects (S11, S14) that reported stable stimulus percepts in all of the four sessions exhibited highest C-index values (1.0) of STM type coefficient and significantly nonzero STM type coefficients in the contralateral responses of (LS-RH)/(RS-LH) components. The mean multiple correlation coefficient was 0.80.

**Figure 7:**
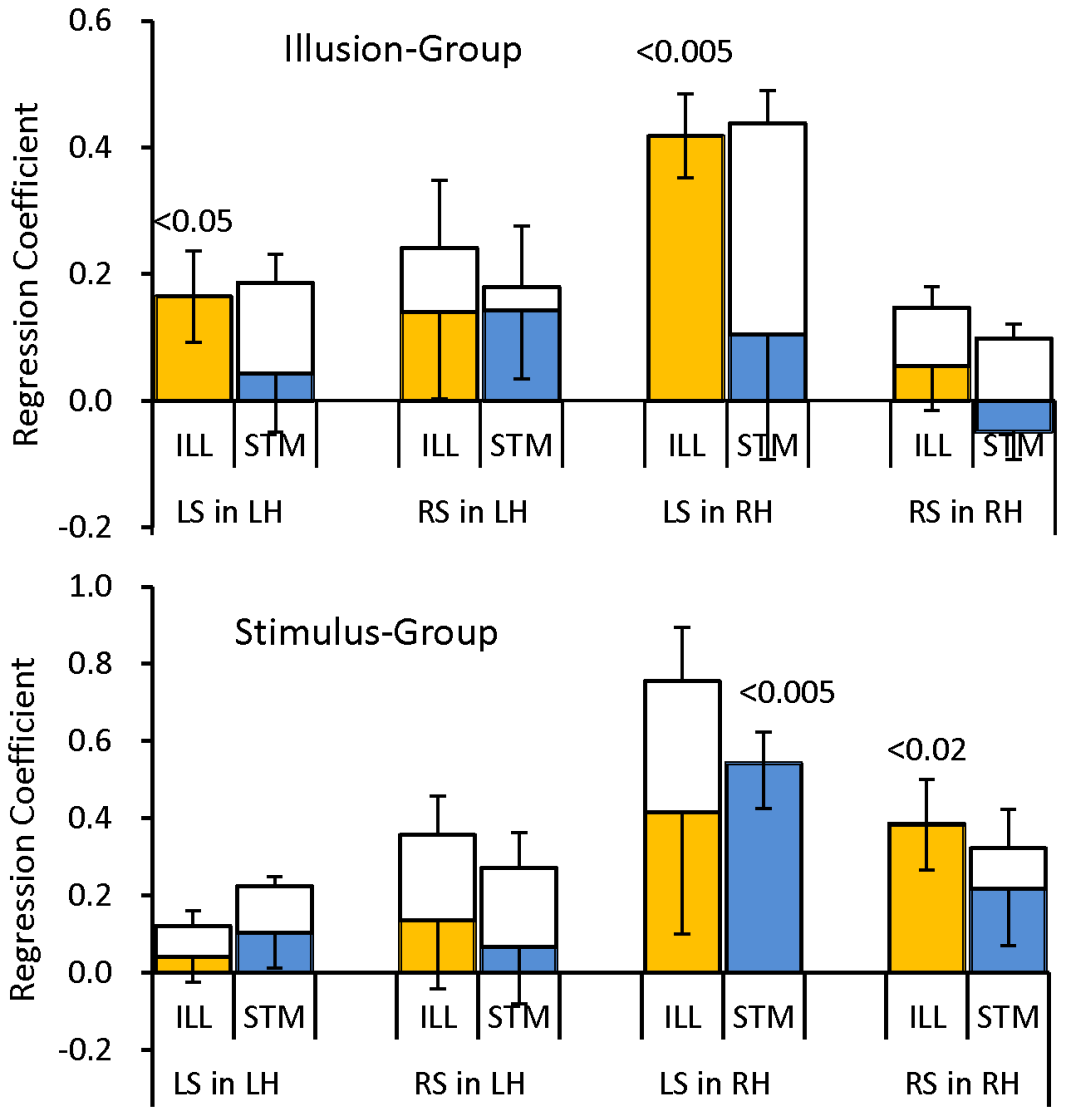
Mean values (color column) and mean of absolute values (white column above zero line) across subjects (n = 6) of ILL type and STM type regression coefficients (indicated below the column) in the response components of LS/RS in the LH/RH. Note the difference in the vertical scale between ILL and STS groups. SE bars are indicated in positive and negative directions for the absolute mean and the mean, respectively. P-values indicate the significance level above zero in the t-test.

Fig. 7 shows the absolute mean of ILL type and STM type coefficients (white columns above zero line) across subjects in the LS/RS-LH/RH responses. Here, the absolute mean is the average of absolute values of those coefficients. Three-way ANOVA with factors of group (ILL/STS), coefficient type (ILL/STS) and response component (LS/RS-LH/RH) indicated a significant main effect of the component (p<0.0000, F(3,30) = 29.6) and interaction of the group and component (p<0.03, F(3, 30) = 3.49). Post hoc multiple comparisons revealed that the coefficients of the LS response in the RH were significantly greater than the coefficients of any other responses in the RH and LH (p<0.0001, Ryan's method), and a simple main effect revealed that the STM group had significantly greater coefficients of LS and RS responses in the RH than the ILL group (p<0.001, F(1,40)≥12.3). These differences are in line with the corresponding differences in the standard deviation of the mean ASSR magnitude; see the resemblance of the SDs in Fig. 4 to the absolute means in Fig. 7. Thus the magnitude of the coefficients replicated the deflection of the ASSR magnitude among stimulus tones. Multiple comparisons further revealed that the coefficients of the LS response in the RH were greater than the coefficients of any other responses in the RH and LH (p<0.0001, Ryan's method).

Also shown in Fig. 7 are the mean values of ILL type and STM type coefficients (color columns) across subjects. Three-way ANOVA revealed only a main effect of component (p<0.02, F(3,30) = 4.21). Post hoc multiple comparisons showed the same difference as in the absolute mean that the LS-RH response was greatest among all responses. Thus, the right hemisphere ASSR to the contralateral tones was the major component. In this component, the mean ILL type coefficient in the ILL group (p<0.005, t(5) = 6.30) and the mean STM type coefficient in the STM group (p<0.005, t(5) = 4.63) were significantly above zero, while the mean STM type coefficient in the ILL group (p>0.25, t(5) = 0.53) and the mean ILL type coefficient in the STM group (p>0.1, t(5) = 1.32) were non-significant.

## Discussion

### Multiple regression analysis

The time course of the mean magnitude of ASSR across subjects as a function of tone number (Fig. 5) seemed to be a mixture of illusion type and stimulus type variations. We obtained quantitative contribution of the two types of variation from the partial regression coefficients of multiple regression analysis. This analysis for individual subjects had some advantages over the analysis of the grand mean ASSR in that statistical evaluation across subjects was possible and that different time courses of high-pitch and low-pitch illusion type variations, which are highly correlated (γ = −0.97) with each other, could be analyzed in separate subjects. The ASSRs of these time courses would otherwise be cancelled out in the mean ASSR across subjects. For the subjects who reported unambiguous percepts of illusion or stimulus in all of the four sessions, we observed significant regression coefficients and the highest consistency-index values of illusion or stimulus type that was consistent with their stable perception. These results support the validity of multiple regression analysis to analyze the ASSRs in terms of illusion and stimulus type variations.

### Neural representation of scale illusion

Important findings from the multiple regression analysis are that the group who reported illusion percept exhibited significant illusion type variation of ASSR, while the group who reported stimulus percept exhibited both significant stimulus and illusion type variations. It should be noted here that the data analyzed for the illusion-group subjects and stimulus-group subjects were provided from the illusion perceived and stimulus perceived sessions, respectively. Given the MEG response as directly reflecting the neural activity generated by intracellular currents, we interpret that the percept of illusory pitch sequence in the illusion group is represented as the time course of the amplitude of ASSR that is characterized by the illusion type variation. That is, the pitch sequence of stimulus tones was encoded as it was perceived, not as it was presented, in the cortical activity of the illusion group. The coexistence of time courses of stimulus type and illusion type variations in the stimulus group suggests that the pitch sequence of stimulus tones was encoded as it was presented in the cortical activity of those subjects, in addition to the activity representing illusory pitch sequences that were not reported as perceived.

Deutsch described [1] that the perception of smooth pitch-contour from competing dichotic tone-sequences in the scale illusion is a result of grouping by frequency range, which integrates the dichotic sequences into high- and low-pitch streams. In this regard, it is known that rapidly alternating sequential tones of ABAB–, differing in pitch, are segregated into two parallel streams of AA– and BB– when the pitches of two tones are well separated. An animal study on the auditory stream segregation [16] has revealed automatic physiological mechanism of low level processing, which is in general accord with the characterization of the segregation as a primitive perceptual phenomenon [31]. A study on human ERPs [32] has suggested that automatic segregation serves as the initial grouping of acoustic elements of AB tones, being independent of attention, and that integration of sequential tones within streams of A and B tones over several seconds occur subsequently in an attention-dependent process. Here, grouping of sequential short-tones by frequency cue is a common feature of auditory stream segregation and scale illusion. Deouell et al. [33] have shown that patients suffering from left auditory extinction, i.e., unconsciousness of left side stimuli, following right hemisphere damage including the auditory cortex are susceptible to scale illusion. From these results of previous auditory segregation and lesion studies, we infer that automatic low-level processing that is independent of conscious attention is involved in the neural mechanism of the scale illusion. As for the duplicated perception of illusion and stimulus type pitch contours in the stimulus group, we presume that those subjects could discriminate stimulus pitches in the right and left ears by intentional listening, while the illusory-pitch streams were perceived simultaneously in an unconscious manner.

The observation that the ASSR contained the pitch sequence of illusory percept suggests that the location of neural correlates of the scale illusion is at or below the site of the ASSR source. Previous MEG studies on ASSR have consistently indicated current dipole sources, which represent intracellular currents as the generator of MEG, in or near the primary auditory cortex (A1) in the superior temporal plane [23], [24], [25], [26]. For the ASSR elicited by sequential short AM tones, MEG sources have been confirmed in the subject's MR image around the A1 in Heschl's gyrus [29]. Such ASSR sources of MEG are in line with the notion of automatic low-level processing of scale illusion. The susceptibility to scale illusion of patients with impairment of the right auditory cortex [32] suggests that coupling of the left and right sounds exists below the level of the auditory cortex.

### Binaural interaction

Given that the time course of ASSR replicated the pitch sequence of illusory percept in the illusion-perceiving subjects, interaction of the tones delivered to the right and left ears should have taken place for the modification of the ASSR. Coupling of the right and left stimulus sounds in the central auditory system has been studied in binaural beat: an oscillatory response of the difference pitch between the two sounds having slightly different frequencies. The binaural beat in animals was found at interaural frequencies of 20–63 Hz occurring in the superior olivary complex [34] and at 1–10 Hz in the inferior colliculus [35] of the brainstem. Binaural responses in humans have been detected as 40 Hz ASSR of EEG [36] and MEG [37] and as low-frequency (3–7 Hz) beats of MEG [38] and ERP [39]. Here, the low frequency MEG response was suggested in [38] to provide a neural correlate to the human sensation of beats and also to reflect higher order process related to subjective beat-fluctuation. In addition to the beat responses, enhancement or suppression of evoked potentials was observed for the stimulation of binaural clicks in the medial geniculate body (MGB) of the thalamus in guinea pigs, depending on the subdivisions of the MGB from which the response was recorded [40]. The authors also suggested that the pattern of binaural responses observed in the primary and non-primary auditory cortices may be processed and encoded at the thalamic level. Auditory evoked potentials in humans have been shown to be reduced in amplitude for the binaural stimulation relative to the sum of monaural responses [41]. Likewise, suppression of MEG response was observed by binaural stimulation, being stronger in ipsilateral than contralateral response [42].

These animal and human studies indicate that the contribution to binaural interaction of auditory inputs exists in the brainstem and thalamus, where low frequency beat and suppression/enhancement of evoked responses are likely to occur in the MGB of the thalamus. Those responses are projected to the auditory cortex and can be detected as MEG response. The observed modification of the time course of ASSR in this study occurred in a rate of a few Hz, not at 40 Hz of modulation frequency, which is given by the tone duration, i.e., tone alternation time, of 745 ms. We thus speculate that the binaural interaction involved in the scale illusion resides in the MGB of the thalamus and possibly in the A1 of the auditory cortex. Both structures have dense bidirectional connections and subserve salient auditory information processing of tuned frequencies [43]. There exist different types of binaural columns in the A1, which are classified as contralateral, ipsilateral and equidominant. Binaural responses of the cells in these columns are mostly additive, i.e., summation, and suppressive [44], [45]. Such binaural interactions might play a role in modification of the ASSR in the scale illusion. For example, responses of cells of the contralateral column, having a best frequency of the left sound, would be suppressed by or added with the right sound because of broad tuning curve of the auditory cortical neurons. In such case, the response to the right sound could be appreciable amount in the output of these column cells, which may contribute to the generation of an illusory response to a switched tone from the left sound to right sound in the left sound response in the right hemisphere, such as the dominant response in this study.

### Hemispheric dominance

Three-way ANOVA revealed that the mean value of regression coefficients across subjects was significantly greater in the right hemisphere response to the left sound than any other response components (Fig. 7). In this response, illusion and stimulus type coefficients were significantly nonzero in the illusion and stimulus groups, respectively. These results suggest that the right hemisphere was functionally dominant in the neural processes, in which modification of the pitch sequence of stimulus tones into a smooth pitch contour takes place, during the perception of scale illusion. The magnitude (absolute-mean) of the regression coefficient was also significantly greater in the right hemisphere response to the left sound than any other response components. In the experimental procedure, we equalized the SPL of stimulus sounds to the right and left ears, but this does not necessarily means that the loudness perceived by subjects was equalized between the two ears. The hemispheric dominance of the auditory response could arise from the loudness difference that might be caused by the variation of ear canal across subjects or other experimental factors. This possibility, however, can be neglected because the greater regression coefficients in the right hemisphere were observed in the contralateral response to the left sound, whereas the coefficients in the ipsilateral response to the left sound in the left hemisphere were not significantly greater than the ipsilateral response to the right sound in the right hemisphere. Thus, the observed hemispheric difference seems to be functional, which is in line with the dominance of the right auditory cortex in the processing of spectral change while the left auditory cortex is more devoted to temporal processing [46], [47]. More specifically, the central region of the spectral processing is the belt cortex neighboring the core area of A1 [48]. This hemispheric specialization and the results of this study apparently contradict the left hemisphere contribution to the susceptibility of illusion, i.e., the intact left hemisphere, of the patients in Deouell et al.'s study [33]. However, activity of the right auditory cortex may not be a necessary condition for the scale illusion. Given the assumption that binaural interaction takes place at the level of the MGB and/or the primary auditory cortex, ipsilateral and contralateral auditory inputs are projected in each side of the MGB and/or in binaural columns in the A1 of each hemisphere. Then the right or left auditory cortex of patients, whichever side is spared, could work in the process of illusion perception.
